# Cogs in the autophagic machine—equipped to combat dementia-prone neurodegenerative diseases

**DOI:** 10.3389/fnmol.2023.1225227

**Published:** 2023-08-31

**Authors:** Sholto de Wet, Rensu Theart, Ben Loos

**Affiliations:** ^1^Department of Physiological Sciences, Stellenbosch University, Stellenbosch, South Africa; ^2^Department of Electric and Electronic Engineering, Stellenbosch University, Stellenbosch, South Africa

**Keywords:** autophagy, mitochondrial function, lysosomes, mitophagy, neurodegenerative diseases, proteostasis

## Abstract

Neurodegenerative diseases are often characterized by hydrophobic inclusion bodies, and it may be the case that the aggregate-prone proteins that comprise these inclusion bodies are in fact the cause of neurotoxicity. Indeed, the appearance of protein aggregates leads to a proteostatic imbalance that causes various interruptions in physiological cellular processes, including lysosomal and mitochondrial dysfunction, as well as break down in calcium homeostasis. Oftentimes the approach to counteract proteotoxicity is taken to merely upregulate autophagy, measured by an increase in autophagosomes, without a deeper assessment of contributors toward effective turnover through autophagy. There are various ways in which autophagy is regulated ranging from the mammalian target of rapamycin (mTOR) to acetylation status of proteins. Healthy mitochondria and the intracellular energetic charge they preserve are key for the acidification status of lysosomes and thus ensuring effective clearance of components through the autophagy pathway. Both mitochondria and lysosomes have been shown to bear functional protein complexes that aid in the regulation of autophagy. Indeed, it may be the case that minimizing the proteins associated with the respective neurodegenerative pathology may be of greater importance than addressing molecularly their resulting inclusion bodies. It is in this context that this review will dissect the autophagy signaling pathway, its control and the manner in which it is molecularly and functionally connected with the mitochondrial and lysosomal system, as well as provide a summary of the role of autophagy dysfunction in driving neurodegenerative disease as a means to better position the potential of rapamycin-mediated bioactivities to control autophagy favorably.

## 1. Introduction

**Figure 1:**
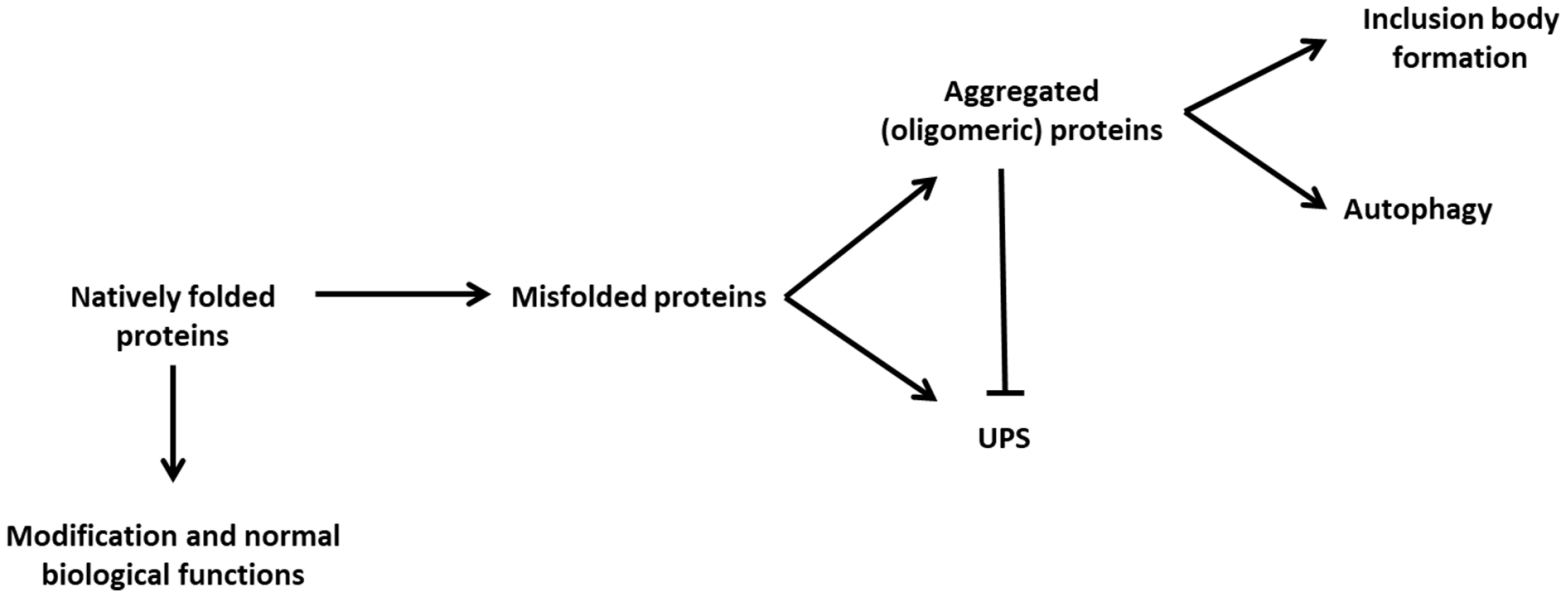
The proteostatic balance through cellular degradation strategies. Natively folded proteins usually undergo modification and processing contributing to normal biological function. However, natively folded proteins may suffer events resulting in their misfolding. The UPS is capable of degrading monomeric misfolded proteins, however, misfolded proteins may become aggregated and unable to be degraded by the UPS. As such autophagy becomes the primary means of maintaining proteostasis by degrading aggregated proteins. These proteins may continue to aggregate to the point at which they cause cellular stress and reduce the ability of autophagy to maintain the proteostasis of the cell. Large, aggregated protein clusters become insoluble and form deposits within the different regions of the cell, depending on the particular disease, rather than undergoing degradative events.

Neurodegenerative diseases such as Alzheimer’s disease (AD), Parkinson’s disease (PD), and Huntington’s disease (HD) are progressive in nature and often consist of both inherited as well as sporadic forms (DiFiglia et al., 1997; Baba et al., 1998; Näslund et al., 2000; Meredith et al., 2002; Le and Appel, 2004; Li et al., 2004; Orr et al., 2008; Genuis and Kelln, 2015). Within the context of an increased aging population, as is the case today, the incidence of sporadic AD and PD cases has been seen to be much greater than those of the genetic variants (Alzheimer’s Association, 2019, 2022). The loss of cognitive function is an overlapping feature of each of these diseases. Additionally, each disease is histologically characterized according to the dysfunction of a particular protein that leads to the formation of inclusion bodies that consist primarily of these proteins within regions of the brain that correlate with the symptoms of the disease in question. For example, AD is characterized by the formation of senile plaques that present with β-amyloid deposition in the cerebral cortex (Braak and Braak, 1991), PD by the formation of Lewy bodies containing α-synuclein in the neurons of the substantia nigra (Spillantini et al., 1998) and HD by intranuclear inclusions containing of mutant huntingtin protein (Yu et al., 2003). Prior to the manifestation of a molecular pathology and symptoms these proteins form a necessary part of normal brain physiology, however, there is an increased production of their misfolded forms, which are aggregate-prone, leading to the eventual formation of inclusion bodies (Chiti and Dobson, 2009; Pechmann et al., 2009). Misfolded proteins have been shown to be a fact of life but are quickly cleared before they are produced to such an extent that they outnumber the presence of the native protein forms (Swart et al., 2014; Sweeney et al., 2017). This clearance is maintained by degradative systems such as the ubiquitin proteasomal system (UPS) and macroautophagy (Bence et al., 2001; Komatsu et al., 2006; Tanaka and Matsuda, 2014). However, it is known that the UPS becomes overwhelmed and ineffective when there is an overproduction of aggregated proteins, as is the case in neurodegenerative diseases, leading to a greater dependence of the system upon autophagy as a means of clearance (Figure 1). Although the components are well known in isolation, the interplay between protein cargo and its solubility state, mitochondrial health and lysosomal dysfunction appears central to the development of neurodegenerative diseases. In this review we will discuss the mechanisms in place for protein quality control; particularly macroautophagy, as well as the cellular dysfunction that impacts mitochondria and lysosomes which appear to be at the heart of neurodegenerative disease pathogenesis.

## 2. Protein quality control

Proteostasis, that is the maintenance of protein homeostasis by controlling the abundance, conformation, binding interactions, and distribution of proteins by means of synthesis, degradation or modification is an integral part of normal metabolic functioning of a cell (Balch et al., 2008). Indeed, due to the important roles that proteins play in cellular processes such as signaling, the immune response, and structure, changes to proteostasis may result in cellular dysfunction and detrimental effects such as ER stress and autophagy dysfunction (Chen et al., 2001; Mcnaught et al., 2002; Chiti and Dobson, 2006; Hara et al., 2006). Therefore, there is a clear need for this balance to be carefully maintained for the sake of cell survival. Two degradative pathways exist and are used by the cell to ensure that proteostasis is maintained.

The first degradative pathway is the ubiquitin-proteasomal system (UPS) which is critical for the rapid degradation of short-lived, monomeric proteins (Kraft et al., 2010). The ubiquitin tagging system component is responsible for covalently binding cytoplasmic entities with ubiquitin, which acts as the recognition motif for the 26S subunit of the proteasome. Thus, the ubiquitination system provides the UPS with a high degree of specificity. It is however possible for the proteasome to become overwhelmed by substrates present (Bence et al., 2001; Mcnaught et al., 2002; Rideout et al., 2004; Pandey et al., 2007; Wang et al., 2020). This occurs when protein targets express irregular structures, resulting in misfolded conformations which are hydrophobic and aggregate-prone. As these proteins aggregate, they from larger clusters and are unable to pass through the proteasome (Johnston et al., 1998). In this instance, the proteostatic balance has been disrupted and the potential for the formation of insoluble structures such as aggresomes or inclusion bodies has been increased. This disruption leads to a proteostatic shift in which the system becomes more reliant upon autophagy as the means to degrade proteins.

Autophagy is the second key degradative pathway operating in cells. Three types of autophagy exist, namely microautophagy, macroautophagy and chaperone-mediated autophagy. Although each of these are well distinguished from one another, they share the function of delivering cytoplasmic components to lysosomes which contain acidic hydrolases; thereby aiding in degradation of these recruited components. Macroautophagy (hereafter autophagy) is the most well studied of its variants and morphologically distinct from the other forms due to the use of vesicular structures known as autophagosomes to shuttle cytoplasmic components to lysosomes, thereby facilitating degradation (Jahreiss et al., 2008; du Toit et al., 2018a). Autophagy has been shown to display pro-survival functions in cells following metabolic perturbation and cellular stress conditions (Mizushima et al., 2004, 2008; Ravikumar et al., 2010; Sumpter and Levine, 2010). Its degradative capabilities have been shown to extend to many cytoplasmic components, including proteins, mitochondria, peroxisomes and even micro-organisms.

In the past, autophagy was described as a non-selective form of degrading cytoplasmic components for the purpose of generating amino acids to be used in ATP production. Although this is true given nutrient-poor conditions (Sahani et al., 2014), it is now known that autophagy also controls selective cargo degradation given imbalanced homeostasis; including disruption in mitochondrial, lysosomal and ferritin homeostasis (Galluzzi et al., 2017). This selective targeting of cytoplasmic components as cargo is accomplished by proteins that act as receptors of ubiquitinated elements, effectively enabling the distinction between different types of disruptive components and targeting them to the autophagosome for subsequent degradation by lysosomal hydrolases (see section 6). Should the synthesis of misfolded proteins and aggregated proteins outweigh the synthesis and presence of fully functional proteins, proteostasis will be disrupted (Figure 1). Misfolded proteins have been shown to interact with other components of the cell such as mitochondria and the endoplasmic reticulum (ER), disrupting their function and further contributing to disrupted conditions (Lasagna-Reeves et al., 2011; Fang et al., 2019; Sharoar et al., 2019; Evans and Holzbaur, 2020). It becomes crucial therefore for the presence of these proteins to be minimized to preserve the viability of the cell.

## 3. Autophagy flux

A basal level of autophagy is constantly present within the cell and serves a “housekeeping” function; eliminating old or damaged cellular components that would otherwise disrupt homeostasis (Mizushima et al., 2004; Alers et al., 2012; Sarkar, 2013). However, in the event of a disruption in cellular homeostasis due to, for example, starvation or increased misfolded protein production, the rate of clearance through autophagy will need to increase (Yang and Klionsky, 2010; Kim et al., 2011).

**Figure 2:**
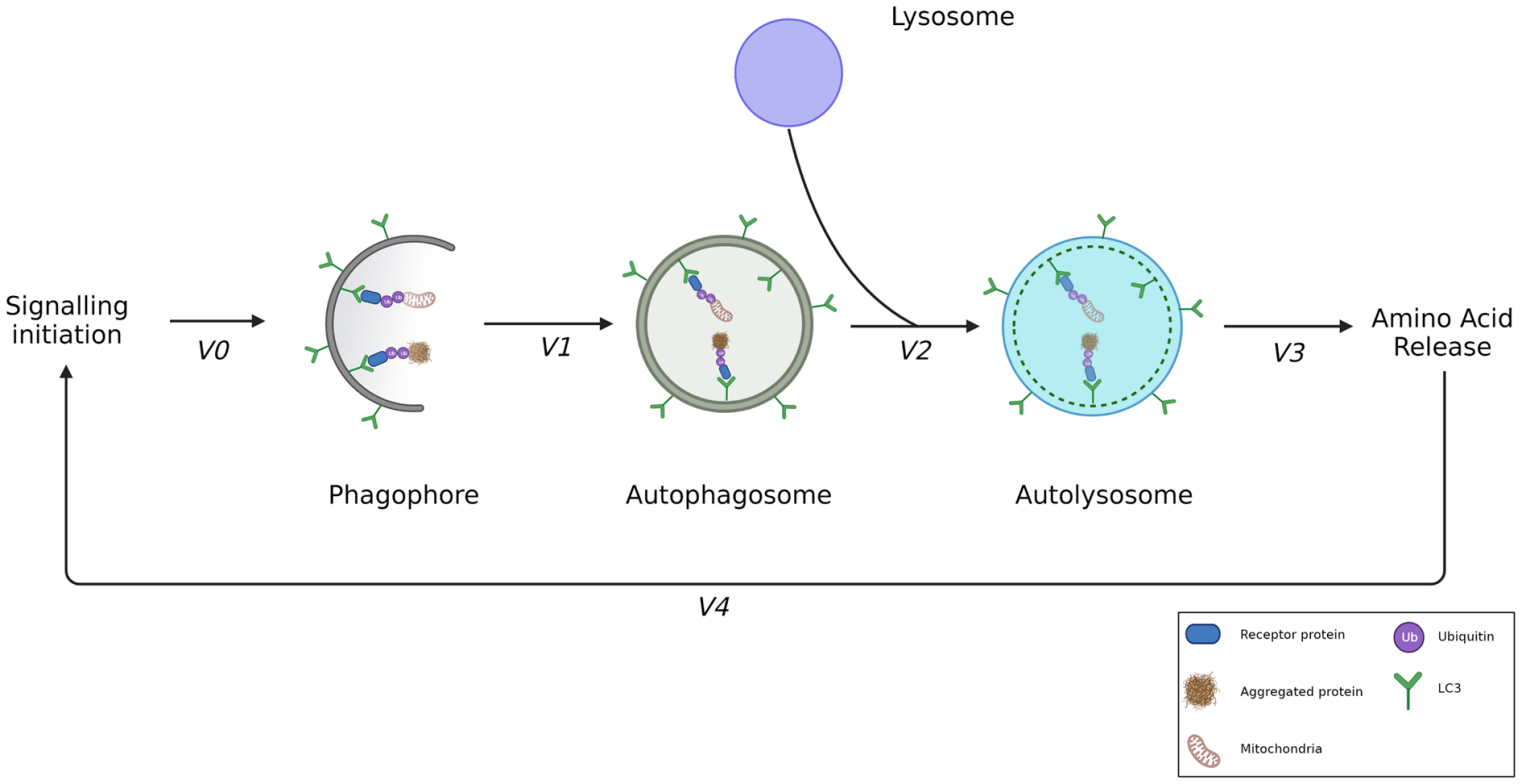
The step-wise rate of autophagosome flux. Signaling events result in the biogenesis of phagophores and lysosomes. Phagophores are decorated by LC3-II, enabling receipt of ubiquitinated cargo such as depolarized mitochondria or aggregated proteins. The formation rate of an autophagosome from a phagophore can be described by measuring V1, enclosing the recruited cargo. V2 however, is the best means of measuring autophagosome flux as it demonstrates the rate of autophagosome degradation by the formation of autolysosomes. V3 demonstrates the rate of amino acid release following the degradation of cargo. V4 shows the feedback of amino acids exerted on the initial signaling event. Adapted from Loos et al., (2014).

Autophagy can be characterized as a step-by-step process (Figure 2) which begins with the initial synthesis of the phagophore which then matures into an autophagosome; the double-membrane vesicle that characterizes the pathway (Loos et al., 2014). Once it has sequestered cargo, the autophagosome is transported to and fuses with a lysosome, resulting in an autolysosome, which ensures the degradation of cargo. The term ‘autophagy flux’—the rate of protein degradation through the autophagy pathway—can be used to quantitatively describe the overall degradative activity and capacity of the pathway (Klionsky et al., 2016). Given conditions such as starvation or increased misfolded protein load, it becomes necessary for the autophagy flux to be increased. Indeed, given such conditions it has been observed that the cellular system will increase the number of autophagosomes thereby effectively increasing the autophagosome pool size available for cargo sequestration and delivery (Boland et al., 2008; du Toit et al., 2018b; de Wet et al., 2021).

As the autophagy pathway involves the participation of many molecular role players, including autophagosomes, lysosomes and cargo, it becomes necessary to discern which of these are being used to measure and define autophagy flux. The term “autophagosome flux” has been proposed and is defined as the rate of flow along the vesicular pathway. The techniques used to measure it infers a distinction between the vesicular—that is, the molecular machinery that make up the autophagosome—and the cargo flux (Loos et al., 2014). This is of importance as not all proteins are degraded at the same rate nor does autophagy occur at the same rate in every tissue type. Indeed, whilst there may be indications of high autophagic activity—such as large autophagosome pool sizes— the inherent degradation rate of proteins may differ according to different tissue types or even brain regions (Mizushima et al., 2004; Lumkwana et al., 2017). Additionally, we have previously shown that an increase in autophagosome pool size does not necessarily infer an equal or proportional increase in the cargo receptor abundance and availability (de Wet et al., 2021). Hence, a distinction between the cargo clearance and autophagosome flux as separate entities may be necessary. To measure autophagosome flux one must consider the total number of autophagosomes within the cell at a time point and contrast this with the number of autolysosomes at the same time, in the absence and presence of an autophagosome/lysosome fusion inhibitor or lysosomal deacidifying agent, such Bafilomycin A1 or chloroquine (Li et al., 2013; du Toit et al., 2018a). In doing so, it becomes possible to measure the change in the number of autophagosomes, lysosomes and autolysosomes, respectively, that are present in the cell within a given time frame and thereby reveal their relationship in contributing toward the degradative potential of the cell. For this reason, autophagosome, lysosome and autolysosome pool sizes should be measured simultaneously to better understand the autophagosome flux, while measuring both the autophagosome and cargo flux may enable greater insight into the clearance capabilities of the pathway.

Indeed, the step-by-step nature of the autophagy pathway allows a greater number of parameters to be assessed to describe the degradative activity of the system. The following sub-sections will be used to discuss the control and stages of autophagy flux as separate entities such that it is clearer what factors may influence the degradative capacity of autophagy (Figure 2).

## 4. Autophagy control

### 4.1. mTOR-dependent induction – mitochondrial and lysosomal enhancement of autophagy

**Figure 3:**
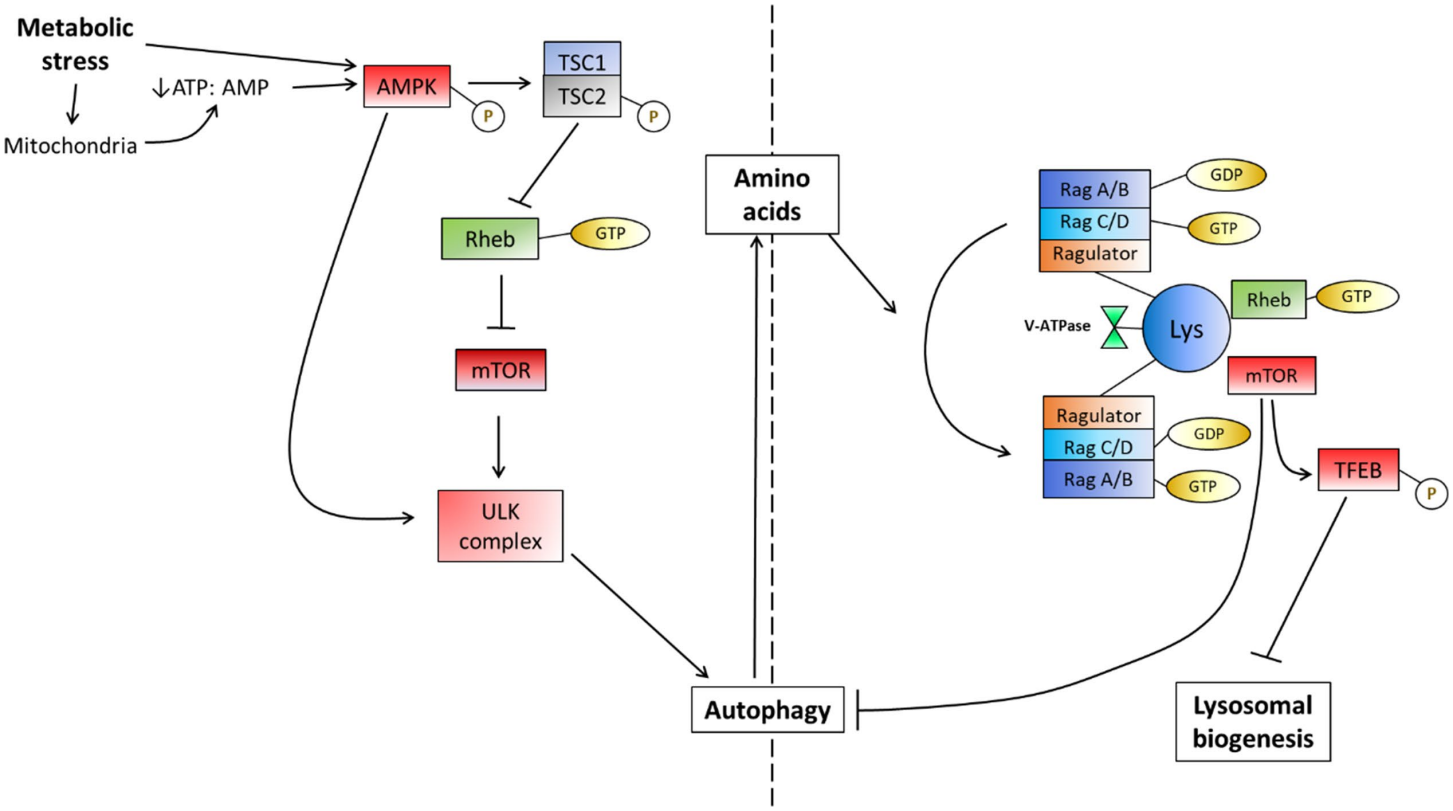
The mTOR-dependent response to metabolic stress in the form of starvation. Metabolic perturbations encompass many potential factors which often lead to a response directed toward the mitochondria. This may cause the depletion of ATP due to its actions on mitochondria or may be directly sensed by AMPK to induce autophagy by inhibiting mTOR. AMPK may also directly dephosphorylate the ULK complex to induce autophagy independently of mTOR, however, this often occurs at lower levels. The availability of amino acids is additionally sensed by the v-ATPase on the lysosome membrane and causes a change in the Rag complex activation, recruiting mTOR and thereby driving an inhibition reaction on autophagy when amino acids become available [Adapted from Sarkar (2013)].

The autophagy pathway is well regulated such that the basal flux becomes rapidly increased given the introduction of a metabolic stressor or metabolic perturbation. The pathway that regulates the induction of autophagy is comprised of a complex network of proteins that integrate various factors and so impact autophagy flux (Figure 3). Since mitochondria require amino acids to preserve oxidative phosphorylation activity as well as glucogenic and ketogenic amino acids as additional substrates to drive ATP synthesis, the role of AMP-activated protein kinase (AMPK) has been closely tied to mitochondrial requirements and health (Hardie et al., 2012; Rabinovitch et al., 2017). Moreover, the ability of the AMPK machinery to sense ATP levels and hence energetic charge of the cell and to regulate autophagy accordingly demonstrates a close molecular link between mitochondrial health, the energy status of the cell and autophagy activity. Furthermore, the control of AMPK over autophagy induction becomes of great importance when one considers that autophagy is capable of providing amino acids that are used for mitochondrial respiration (Kim et al., 2011; Burman et al., 2017). When AMPK senses a lack of ATP, it becomes phosphorylated (Hardie et al., 2012; Rabinovitch et al., 2017) and subsequently phosphorylates the TSC2 (tuberous sclerosis complex) component of the TSC1/2 heterodimer, deactivating it. The result thereof is a downstream inhibition of the mammalian target of rapamycin complex 1 (mTOR). Due to the large degree of signaling-associated input it receives to regulate autophagy, mTOR is often referred to as the master regulator of autophagy (Wullschleger et al., 2006; Boyle and Randow, 2013). mTOR has been shown to receive and integrate stimuli from various signaling pathways in such a way that it aids in cellular repair and growth (Betz and Hall, 2013). AMPK and mTOR have been shown to regulate autophagy in accordance with the metabolic demand of the cell by phosphorylating components of the Ulk1/2-Atg13-FIP200 (ULK) complex: the initiator of autophagosome biogenesis (Alers et al., 2012). Indeed, given starvation conditions, AMPK becomes phosphorylated, subsequently phosphorylating mTOR such that autophagy is induced, as observed by the dephosphorylated state of the ULK complex (Kim et al., 2011).

In addition to the regulation of the ULK complex by AMPK and mTOR, there appears to be a molecular link between the overall abundance of lysosomes per cell and the regulation of autophagosome synthesis initiation (Sancak et al., 2010; Yu et al., 2010; Martina et al., 2012). Studies have shown that transcription factor EB (TFEB); the master regulator of lysosomal biogenesis, plays an important role in regulating the expression levels of autophagy genes. Given starvation conditions, TFEB translocates from the cytoplasm to the nucleus to induce lysosome biogenesis. Additionally, it has been observed that the overexpression of TFEB results in an increased number of autophagosomes, demonstrating a direct autophagy inducing role (Sardiello et al., 2009; Settembre et al., 2011). mTOR has been shown to regulate TFEB activity to avoid excessive levels of autophagy (Martina et al., 2012). To achieve this, mTOR must first be recruited to the lysosome. Lysosome membranes are decorated by amino acid-sensing complexes that consist of Ras-related GTPase-binding proteins (Rags) and Ragulators (Eunjung et al., 2008). Rags exist as heterodimers consisting of either RagA or RagB that is bound with either RagC or RagD; forming a trimeric complex that is collectively known as the Ragulator, and it links the Rags with v-ATPase. Low concentrations of amino acids activate v-ATPase, resulting the enhanced acidification of lysosomes through the hydrolysis of ATP (Sancak et al., 2008; Bordi et al., 2016). When amino acids are sensed, the Rag complex will be activated and causes the translocation of mTOR to the lysosome membrane. Rheb GTPase is a key activator of mTOR activity and its localization to the lysosome membrane is a key regulator of mTOR activity (Tee et al., 2003; Eunjung et al., 2008). Both the AMPK and Rag machinery hence sense the energetic state of the cell and regulate autophagy accordingly, demonstrating the importance of mitochondria and lysosomes in the control of autophagy induction. mTOR is able to carry out its inhibitory actions on TFEB and therefore lysosome and autophagosome biogenesis when Rheb is bound with GTP (Zoncu et al., 2011).

Evidently, mTOR is capable of regulating autophagy through many avenues and has an impact on both autophagosome and lysosome pool sizes. This lends greater control over the degradation of cytoplasmic components, thereby contributing toward effective control over basal autophagy flux in response to metabolic stress. It becomes clear however, that mTOR is not the sole regulator of the autophagy pathway. Rather, the interplay between mTOR, AMPK, TFEB and the Rag complex are key to autophagy signaling and regulation. Indeed, in the case of Alzheimer’s disease, reduced removal of aggregated amyloid-β results in disrupted proteostasis and the manifestation of the neurodegenerative symptoms of Alzheimer’s disease (see section 8.1) (Haass and Selkoe, 2007). Additionally, excessive levels of autophagy flux have been shown to be harmful, with the induction of a specific autophagy-dependent cell death termed autosis, which is triggered when there is a high demand for membrane material to generate autophagosomes, thus resulting in damage to the ER and mitochondria (Kriel and Loos, 2019).

### 4.2. mTOR-independent autophagy control

The activity of mTOR, along with the energy sensing activity of AMPK and the lysosome-associated machinery, allows for the fine-tuning of the autophagy pathway response according to the metabolic needs of the cell. These interactions represent the mTOR-dependent means of autophagosome biosynthesis signaling. There are however mechanisms of inducing autophagy that are independent of mTOR activity. For example, the recruitment of Beclin-1 is crucial as a molecular machinery component during the formation of the phagophore; the pre-autophagosome membrane (Kihara et al., 2001; Pattingre et al., 2005; Russell et al., 2013). Additionally, there is a close interaction between the inositol signaling pathway and calcium presence and abundance (Sarkar et al., 2005; Vicencio et al., 2009) as both work to regulate autophagy induction independent of mTOR. Furthermore, the regulation of acetylation, the post-translational modification that involves the transfer of an acetyl group, has gained increasingly attention due to its role in controlling autophagy flux (Xie et al., 2010). Indeed, components such as sirtuin-1 (Sirt1); a NAD-dependent deacetylase capable of sensing metabolic stress (Haigis and Sinclair, 2010), and E1A-binding protein p300 (EP300); an acetyltransferase (Lee and Finkel, 2009) are two agents that influence the acetylation status of proteins. Both aid in regulating the recruitment of core autophagosome machinery components such as Atg5, Atg7 and LC3 to the phagophore and thus control autophagosome elongation (Bánréti et al., 2013; Pietrocola et al., 2015; Pineda-Ramírez et al., 2020). Indeed, substances such as spermidine, curcumin and resveratrol have been shown to cause changes in autophagy signaling that lead to the increase of autophagy flux by increasing autophagosome biogenesis (Chung et al., 2010; Morselli et al., 2011; Ai et al., 2015; Pietrocola et al., 2015).

## 5. The autophagosome assembly

**Figure 4:**
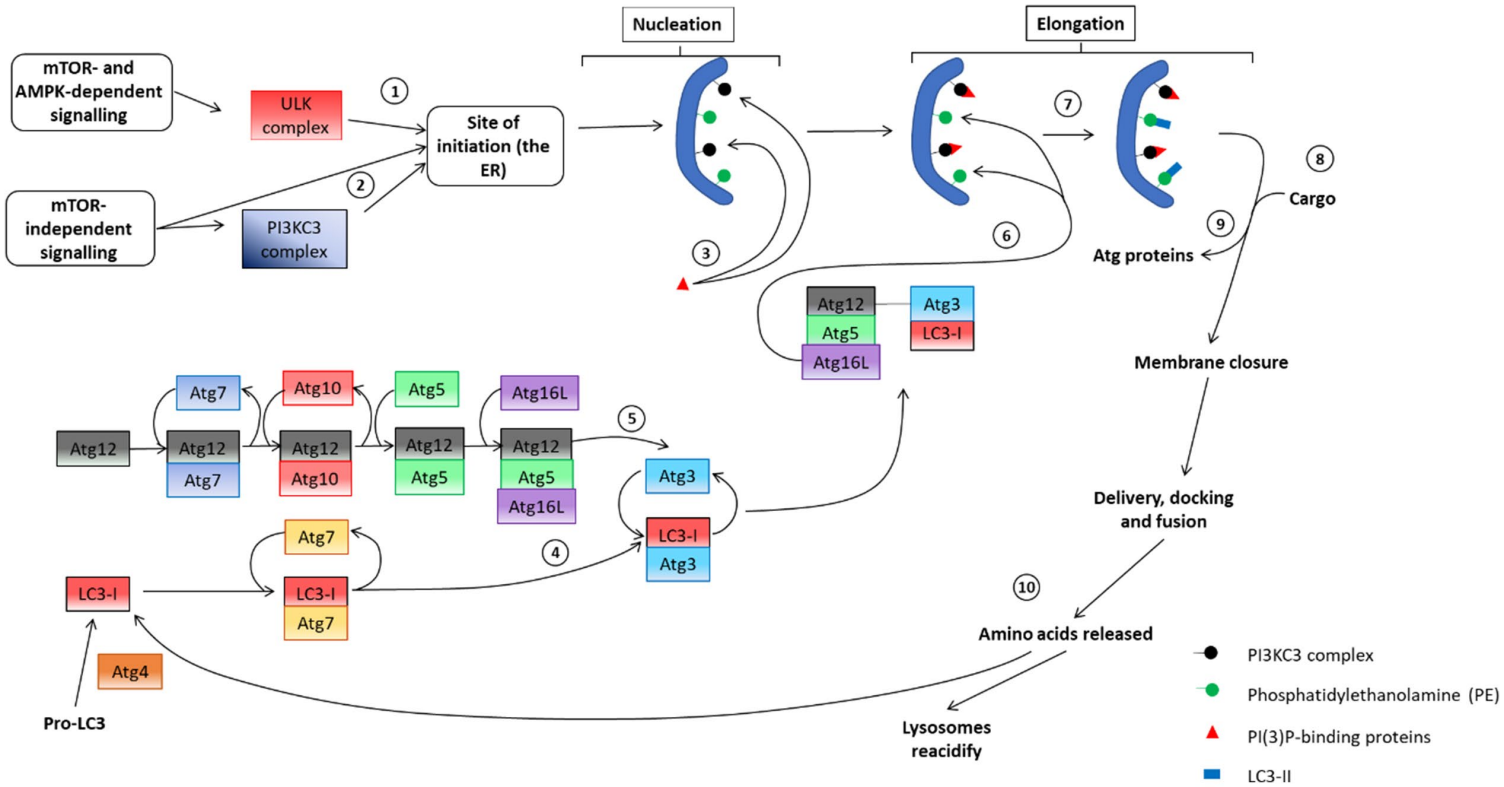
An overview of the autophagosome pathway, including the molecular machinery of each step. (1) Initial mTOR dependent recruitment of the ULK complex to the ER to facilitate the (2) subsequent recruitment of the PI3KC3 complex to the ER to enable phagophore nucleation. (3) PI(3)P-binding proteins aid in the nucleation step and assist in the recruitment of cargo. (4) and (5) depicts the two ubiquitin-like conjugation systems that are in place to assist in phagophore elongation. These proteins engage in the recruitment of the other components to the phagophore membrane. (6) LC3-I interacts with PE on the phagophore membrane, causing a lipidation reaction that produces LC3-II, aiding in the (7) recruitment of cargo to the membrane. (8) Atg proteins dissociate from the autophagosome membrane and aid in membrane closure. Following closure, autophagosomes are considered mature and interact with microtubules to facilitate delivery, docking and fusion with lysosomes to undergo degradation of sequestered cargo. Following degradation, the autophagy system must be reset in preparation for subsequent autophagy events. To this end (9) lysosomes are reacidified and LC3 is recycled under care of the Atg4 protein [adapted from Sarkar (2013)].

The molecular events necessary following the signaling event that led to the generation of a mature autophagosome that contains sequestered cytoplasmic cargo and is functionally able to interact with a lysosome for degradation, is highly complex and comprises of multiple steps (Figure 4). This section will address the autophagosome machinery, that is, the core proteins recruited to the phagophore membrane, leading to the formation of a mature autophagosome. Autophagosome biosynthesis is marked by the initial translocation of the ULK complex to the ER, the site of autophagosome initiation (Koyama-Honda et al., 2013). Once present on the ER membrane, the ULK complex recruits the PI3K complex III (PI3KC3) containing Atg14L (Russell et al., 2013). Once recruited to the ER membrane, PI3KC3 phosphorylates phosphatidylinositol (PtdIns)—a lipid present on the membranes of many organelles, including the ER—to produce PI(3)P (phosphatidylinositol-3-phosphate). In parallel with this event, there is recruitment of proteins that specifically bind to PI(3)P, such as DFCP1 (double FYVE domain-containing protein 1) and WIPI (WD-repeat-interacting phosphoinositide protein). The exact role of these proteins remains unclear; however, it seems clear that their recruitment precedes that of the autophagy-related proteins (Axe et al., 2008; Koyama-Honda et al., 2013; Amaya et al., 2015). Additionally, these proteins contain a FYVE domain, which enables them to bind with the membrane of the ER and possibly the Golgi apparatus and therefore the vesicle derivatives of each. As such, PI(3)P-binding is considered to form a scaffolding matrix on the phagophore that facilitates the recruitment of autophagy-related (Atg) proteins to the phagophore after the nucleation event.

Before an autophagosome can sequester and deliver cargo, it requires modification from two ubiquitin-like conjugation systems to facilitate its elongation (Figure 4). First, Atg12 is conjugated to Atg5, which occurs due to a reaction with Atg7 and then with Atg10. Next, Atg16L is non-covalently conjugated to the Atg12-Atg5 complex, resulting in the formation of the Atg12-5-16 L complex (Mizushima et al., 2003; Tanida et al., 2004). The complex is then targeted toward phosphatidylethanolamine (PE), another membrane lipid located on the pre-autophagosome membrane (Mizushima et al., 1999).

The second conjugation system involves the transfer of microtubule-associated protein 1 light chain 3 (LC3). This process begins with the cleavage of pro-LC3 by Atg4 resulting in LC3-I. LC3-I is subsequently conjugated with Atg7 and then with Atg3. The interaction between Atg12 and Atg5 enables Atg12 to sequester Atg3, removing it and bringing LC3-I into proximity with PE. The reaction between LC3-I and PE results in the lipid-bound LC3-II (Fujita et al., 2008b). The presence of LC3-II marks the end of autophagosomal elongation and autophagosomes are now prepared for cargo sequestration (section 6). Once cargo has been sequestered, Atg proteins dissociate from the autophagosome membrane. This dissociation is thought to enable the closure of the autophagosome (Cebollero et al., 2012). LC3-II remains as part of the autophagosome membrane for the duration of its lifetime and is used as a marker of mature autophagosomes when assessing autophagosome abundance or autophagosome puncta counts (Pankiv et al., 2007; Boland et al., 2008; Jahreiss et al., 2008; du Toit et al., 2018a). Additionally, LC3-II is useful in enabling interaction between autophagosomes and microtubules, acting as an anchor point for molecular motors; proteins that drive the movement of the autophagosome (Fass et al., 2006; Jahreiss et al., 2008; Pankiv et al., 2010). Microtubules play an important role in the maturation of autophagosomes as well as their delivery, docking and fusion with lysosomes (Shen and Mizushima, 2014). The role of microtubule-dependent delivery in the autophagy process will be further discussed in section 7.

After lysosomal-dependent degradation, amino acids are released into the cytosol where they can be used for aerobic respiration by mitochondria (Scherz-Shouval and Elazar, 2007; Rabinowitz and White, 2010). LC3-II is delipidated to LC3-I by Atg4, thereby replenishing the cytoplasmic pool for future autophagy events (Fujita et al., 2008a; Kaizuka et al., 2016). The proteins involved in ensuring functional autophagosome generation and providing autophagosomes with the ability to successfully sequester cargo and interact with microtubules to enable their delivery to lysosomes demonstrate a high level of complexity. The molecular machinery necessary for autophagosome synthesis is of key interest and studies are ongoing to elucidate the precise role of many of these protein during autophagy enhancement and cargo sequestration.

## 6. Cargo specificity – knowing the enemy

Once elongated, the autophagosome is ready to receive cargo that is to be delivered to the lysosome for degradation. This step has received major attention as the autophagosome may either sequester cytoplasmic components as cargo in a non-selective, bulk manner (Mizushima, 2007), or in a highly selective manner (Kirkin et al., 2009; Zhang and Ney, 2009; Deosaran et al., 2013). The understanding of the specificity of autophagy has created a need amongst researchers to differentiate between several subtypes of autophagy according to the specific cargo targets in the context of metabolic or homeostatic perturbation (Galluzzi et al., 2017). The exact mechanism by which cargo recognition is regulated is not clear, however ubiquitinated cargo appears to be an overlapping feature (Kim et al., 2008) which is exploited by proteins bearing a ubiquitin binding area (UBA) domain as well as a LC3-interacting region (LIR). In this way, these receptor proteins, sometimes termed adaptors, are able to bind ubiquitinated components as well as LC3-II, thereby linking cargo with autophagosome machinery for effective cargo sequestration (Pankiv et al., 2007; Fujita et al., 2008a; Figure 2).

One such receptor protein is p62/SQSTM1 (hereafter p62). Due to its various functional domains, p62 has been shown to play an important role in many physiological processes such as cellular signaling pathways, inclusion body formation and tumorigenesis (Moscat and Diaz-Meco, 2009; Lim et al., 2015). In the context of cargo degradation, p62 has been shown to be present in autophagy progression and is therefore useful as a marker of autophagy activity (Ravikumar et al., 2005). Additionally, p62 has been shown to bear a Phox and Bem1 (PB1) domain which enables it to oligomerize with other p62 receptors. In so doing, p62 can self-aggregate along with its cargo to further enhance degradation (Kirkin et al., 2009). Although p62 appears to be involved in many forms of autophagy, its importance lies in its function to serve as a receptor that is primarily associated with aggrephagy; the autophagy-dependent degradation of protein aggregates (Kopito, 2000; Bjørkøy et al., 2005; Caccamo et al., 2017). This role is vital in the context of neurodegenerative diseases in which harmful misfolded proteins are generated and may lead to the formation of inclusion bodies if left unchecked (Komatsu et al., 2007; Salminen et al., 2012; Sarkar et al., 2014).

Another receptor protein, neighbor of BRCA1 gene 1 (NBR1), has been shown play several roles in maintaining cell survival (Mardakheh et al., 2010; Kim et al., 2019; Marsh et al., 2020), including the autophagy-dependent degradation of peroxisomes, known as pexophagy (Deosaran et al., 2013). Furthermore, NBR1 has also been shown to play an important role in aggrephagy as a compensatory receptor given the absence of p62 (Kirkin et al., 2009). Evidence has shown that NBR1 may be cleared through the endosomal rather than the autophagosomal pathway (Mardakheh et al., 2010), however, given that NBR1 bears the LIR and UBA domains necessary for autophagy-associated degradation, it may be plausible that NBR1 is preferentially degraded through the endosomal pathway, but has increased involvement in the autophagy-lysosomal pathway as p62 availability decreases.

Mitochondria are vital in the maintenance of the cell survival. Mitochondria are spread out through the cell as highly dynamic network; undergoing fission and fusion reactions as necessary. Fusing to facilitate efficient oxidative phosphorylation as well as protecting and maintaining mitochondrial DNA and undergoing fission to distribute mitochondria across the cell and segmenting depolarized portions to ensure their subsequent clearance (Parone et al., 2008; Twig et al., 2008; Wang et al., 2009; Perciavalle et al., 2012; Rana et al., 2017). To this end, mitophagy; the autophagy-dependent degradation of mitochondria, is yet another key autophagy subtype. Indeed, failure to eliminate dysfunctional/depolarized mitochondria results in the continued production of ROS, therefore threatening cell viability (Quintanilla et al., 2009; Wu et al., 2011). Several mitophagy receptors have been identified, presumably demonstrating the importance of mitochondrial health and turnover. These include receptors such as BNIP3 and NIX, optineurin (OPTN), optic atrophy 1 (OPA1) and the PINK/Parkin system (Zhang and Ney, 2009; Shi et al., 2014; Marceau et al., 2015; Diot et al., 2018; Padman et al., 2019; Evans and Holzbaur, 2020). The variety of identified receptors for mitophagy is a true testament to the importance of maintaining a healthy mitochondrial network and minimizing oxidative stress produced by dysfunctional mitochondria.

Of the various subtypes of autophagy that exist, aggrephagy is the one most often studied and best characterized. This is due to its critical role in re-establishing disrupted proteostasis, especially when the activity of the UPS has been impeded. Other types of autophagy, such as lysophagy, reticulophagy, xenophagy and several others have also been described along with their potential receptors (reviewed in Galluzzi et al., 2017). It is important to note that the selective nature of autophagy has only recently been identified and investigations into the exact mechanism through which cargo is sequestered are ongoing.

## 7. The molecular broad strokes of dementia-prone neurodegenerative disease

**Figure 5:**
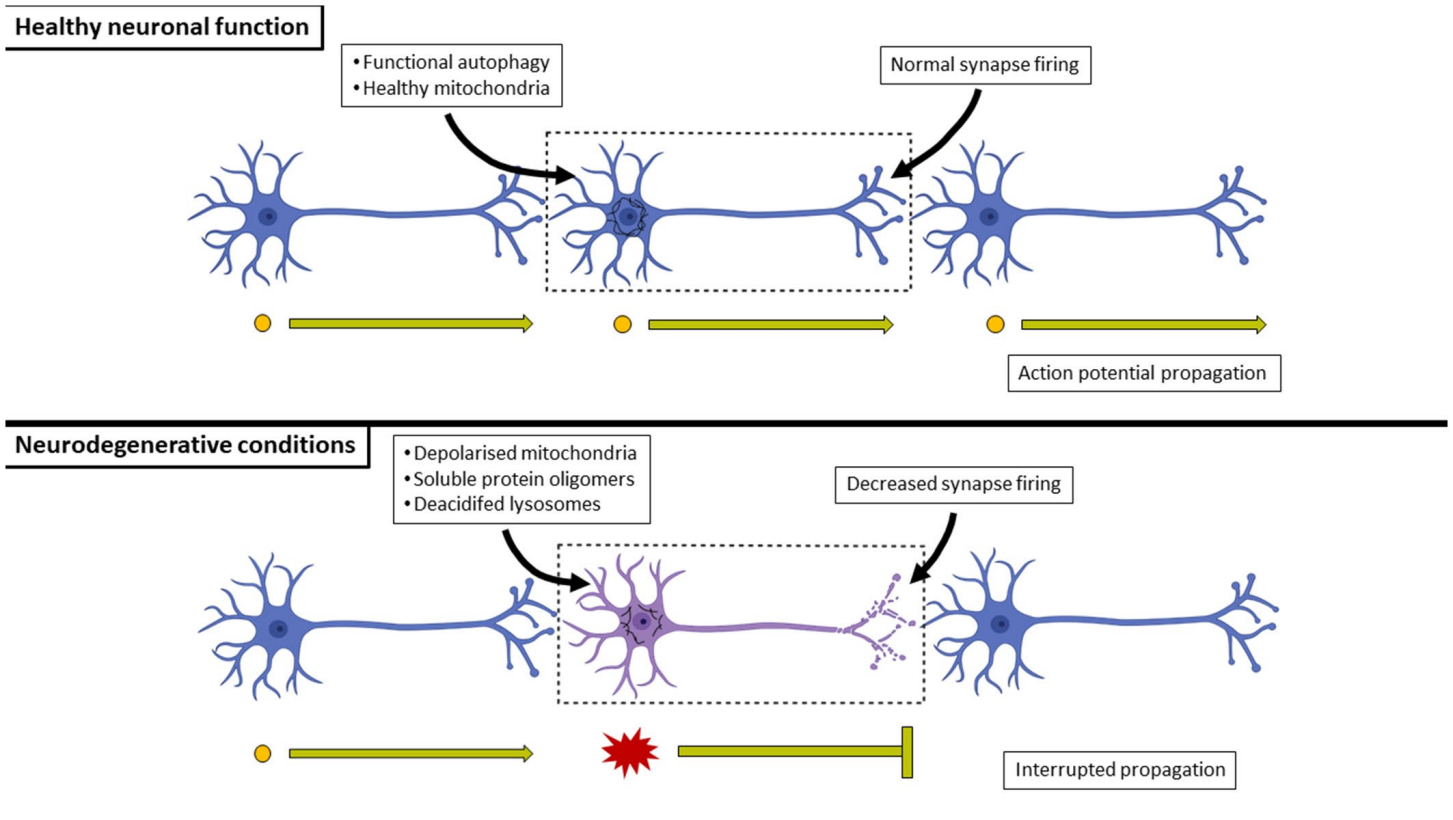
A comparison between healthy and neurodegenerating neuronal connections. In healthy neurons, basal autophagy is at such (typically very high) level that harmful misfolded proteins are minimized and eliminated, preventing the formation of aggregates and the co-localization with mitochondria which would otherwise impair ATP generation. In contrast, the over-production of harmful misfolded proteins will result in their aggregation. Their overproduction may occur at a rate greater than that of the cell’s autophagy flux, effectively leading to their harmful activities of aggregating to the point of forming insoluble inclusion bodies, impairing mitochondrial respiration and leading to a decrease in synaptic activity of the cell. Neurons eventually undergo cell death as a result, effectively breaking circuits.

At a molecular level, neurodegenerative diseases are characterized by the increased presence of soluble oligomers, dysfunctional mitochondria, deacidified lysosomes and ER stress (Figure 5). Which proteinaceous cargo is problematic is what defines the neurodegenerative disease and will be discussed in subsequent sections. Due to the conformation taken on by misfolded protein monomers (Hamano et al., 2008; Chiti and Dobson, 2009), these proteins become increasingly aggregated and the resulting oligomers cause a proteostatic imbalance (Tanaka and Matsuda, 2014; Martínez et al., 2017). Additionally, due to the extent to which these proteins are aggregated, they become unable to pass through the proteasome component of the UPS. Basal autophagy has been shown to be upregulated in response to the accumulation of proteinaceous components, demonstrating a shift in protein degradation that is reliant upon autophagy (Nixon, 2007; Lim et al., 2015).

The ER is crucial in maintaining proteostasis and a disruption of that proteostasis, due to the overproduction of misfolded proteins, has been shown to induce ER stress; a major contributor toward the overall cellular stress that results in increased cytoplasmic calcium (Ca^2+^) (Goussakov et al., 2010; Hetz and Mollereau, 2014; Villegas et al., 2014; Kaushik and Cuervo, 2015; Martínez et al., 2017). Furthermore, the misfolded proteins associated with neurodegenerative diseases have been shown to co-localize with and disrupt the electron transport chain complexes of mitochondria, thereby disrupting neuronal bioenergetics (Manczak et al., 2006; Chinta et al., 2010; Lasagna-Reeves et al., 2011; Shirendeb et al., 2011). This mitochondrial disruption carries a knock-on effect as the lack of ATP generation leads to a decrease in the acidified lysosomal pool size. The loss of lysosomal acidity results in an increase in the number of autophagosomes and a decrease in the numbers of autolysosomes in the neuron as the disease progresses (Lee et al., 2011; Burbulla et al., 2017). The combined effects of increased intracellular Ca^2+^ along with dysfunctional mitochondria lead to an increased production of ROS within the cell, priming it toward death (Kishida and Klann, 2007; Loos et al., 2013). Subsequently, abundant cell death causes interruptions between neuronal connections within specific regions of the brain, thereby contributing to clinical symptoms that manifest in patients with the disease. Additionally, many neurodegenerative diseases, such as Alzheimer’s and Parkinson’s disease, have both genetic as well as non-genetic origins, indicating that these diseases may have familial roots or be a result of lifestyle, environment or aging (Andersen, 2003; Winklhofer and Haass, 2010; Genuis and Kelln, 2015; Kell and Pretorius, 2015; Xu et al., 2016; Colacurcio and Nixon, 2017; An et al., 2018). In short, the molecular pathway through which neurodegenerative diseases are manifested in patients is no simple matter and should be approached in a genetic as well as physiological and biochemical manner to reach a consensus on the best possible treatment approach.

Aggregated proteins continue to accumulate throughout the progression of the disease as the pool size of acidified lysosomes continues to decrease. As a result, there is disrupted cellular energetics and death that leads to decreased synaptic firing and consequently, disrupted neuronal networks (Figure 5). The respective insoluble inclusion bodies are used as histological markers of neurodegenerative disease, especially at late stages where autophagy function has been mostly lost due to the loss of acidified lysosomes and neurons have begun to undergo cell death. The region of the brain impacted by the build-up of these insoluble protein aggregates varies according to the particular disease. However, there are often overlapping clinical symptoms, such as dementia or decreased fine motor capabilities that are common between diseases.

### 7.1. Alzheimer’s disease: autophagy dysfunction with two distinct cargoes

Alzheimer’s disease (AD) is histologically identified by the presence of amyloid-β (Aβ) inclusion bodies in the cerebral cortex (Braak and Braak, 1991). Additionally, brains of AD patients are often shown to contain neurofibrillary tangles (NFTs) consisting of hyperphosphorylated tau filaments primarily within the hippocampal regions (Polito et al., 2014). The resulting clinical symptoms include memory loss, spatiotemporal confusion and mood changes (Alzheimer’s Association, 2019).

The amyloid precursor protein (APP) is a single-pass transmembrane protein that is found at high levels in the brain (Kamenetz et al., 2003). The exact physiological function of APP remains unclear, however its overexpression in cell models suggests that it aids in cell growth and survival as well as contributing toward the expression levels of dendritic spines (Oh et al., 2009; Lee K. J. et al., 2010), whereas knockout models in mice indicate that it plays a role in development and memory (Senechal et al., 2008). APP is most often located on the plasma membrane of neurons where it undergoes two splicing events as part of its normal processing, first by α-secretase and then by γ-secretase (O’Brien and Wong, 2011) to yield Aβ40. Alternatively, APP may be internalized into an early endosome, where it is cleaved at its β-site by β-site-APP-cleaving enzyme 1 (BACE1) and C-terminal product of β-secretase cleavage (βCTF) (Huse et al., 2000). The resulting protein is shorter than the product of α-secretase cleavage and subsequent cleavage by γ-secretase yields Aβ at a length of 42 (Aβ42) rather than 40 (Aβ40) amino acids (Nixon, 2007). Aβ generation is a normal part of brain metabolism as Aβ is generated in low concentrations at normal physiological conditions. Indeed Abramov et al. (2009) have shown that Aβ has a regulatory role in the release of presynaptic neurotransmitters at the hippocampal region. Importantly, the hydrophobic nature of Aβ42 makes it more prone to aggregation than Aβ40 (Iwatsubo et al., 1994; Xiao et al., 2015). The Aβ42 monomers and oligomers are found in senile plaques that characterize cerebral degeneration in AD (Mucke et al., 2000; Nilsson et al., 2013). Soluble Aβ42 oligomers have been shown to cause neuronal damage in two ways. Firstly, soluble Aβ42 can interact with the outer membrane of mitochondria, thereby leading to its co-localization with the electron transport chain (ETC) machinery (Manczak et al., 2006; Hansson Petersen et al., 2008; Cha et al., 2012; Lee J. H. et al., 2022; Lee S. E. et al., 2022). This interaction disrupts mitochondrial energy metabolism and leads to elevated levels of ROS production. Due to the sensitivity of mitochondria to increased oxidative stress, the increased cytoplasmic ROS produced from Aβ-associated mitochondria will trigger a cascading effect that negatively impacts neighboring non-Aβ-associated mitochondria, effectively stressing the cell if not properly resolved though, for example, mitophagy (D'Amelio et al., 2011; Zündorf and Reiser, 2011; Frank et al., 2012; Rao et al., 2014).

Secondly, the increased production of Aβ42 relative to Aβ40 results in a proteostatic imbalance (Yu et al., 2009). This imbalance appears to be linked with dysfunctional Ca^2+^ regulation (Takuma et al., 2005; Kloda et al., 2007) and an increased concentration of intracellular Ca^2+^ is known to cause a release of caspase co-factors from the mitochondria, thereby priming and sensitizing the cell toward cell death by apoptosis (Pinton et al., 2008; Affaticati et al., 2011). Autophagy responds to increased Aβ42 levels as a means of minimizing the harmful effects brought about by the aggregates (Boland et al., 2008; Klionsky et al., 2016; Caccamo et al., 2017). As the Aβ42 yield increases, there is a decreased ATP output by mitochondria due to ETC interference. As lysosomes need ATP to reacidify after degrading cargo, there is a gradual decrease in the number of acidified lysosomes as oligomers are continually produced (Nixon et al., 2005; Yu et al., 2010; Martina et al., 2012; Lee J. H. et al., 2022). Indeed, lysosomal acidification is crucial in the elimination of toxic oligomers, and a loss thereof has detrimental consequences (Lee et al., 2011). As a result, autophagy flux becomes gradually diminished and misfolded proteins continue to aggregate further. Additionally, this diminishment of autophagy activity will lead to the accumulation of dysfunctional mitochondria due to decreased mitophagy (Komatsu et al., 2006; Martín-Maestro et al., 2017; Lee J. H. et al., 2022; Lee S. E. et al., 2022). Failure to clear these dysfunctional mitochondria will lead to the continued production of ROS and subsequent cytotoxicity (Wang et al., 2009).

AD can be classified according to the age of onset. Early-onset AD (EOAD) occurs in patients younger than 65 years, sometimes as young as 30 years, due to mutations in the gene encoding APP as well as the genes encoding for presenilin 1 (PSEN1) or PSEN2, both of which are key components of the γ-secretase complex and therefore result in an increase in Aβ42 if improperly expressed (reviewed in Hernandez-Sapiens et al., 2022). PSEN1 is also responsible for regulating lysosomal calcium homeostasis and therefore the acidity status of lysosomes, making PSEN1 mutations detrimental to lysosomal pH levels (Lee J. H. et al., 2010; Lee et al., 2015). EOAD is a rare form of AD, and represents between 1 and 5% of AD patients (Reitz et al., 2012). Late-onset AD (LOAD) occurs in patients older than 65 years and, although it can be brought about by genetic mutations (Verghese et al., 2011), its causes are more often multifactorial, ranging from traumatic brain injury, diet and even environmental factors (Sarkar et al., 2014; Kell and Pretorius, 2015; Colacurcio and Nixon, 2017; An et al., 2018), with the primary cause being increased age (Alzheimer’s Association, 2019). Regardless of the classification that AD falls under, both share the presence of senile plaques brought about by the overexpression of Aβ42 as well as atrophy of the brain, resulting in the same clinical symptoms as the disease progresses.

In the past, the mechanism by which neurons release aggregated, insoluble Aβ into the extracellular space was poorly understood, however Bhattacharyya et al., 2021 have recently shed light on the role of mitochondrial associated ER membranes in their release (Bhattacharyya et al., 2021). The presence of co-localized Aβ42, p62 and ubiquitin within senile plaques implies that there an attempt at degrading these proteins through autophagy prior to their expulsion from the neuron (Komatsu et al., 2007; Nilsson et al., 2013). It may be the case that these proteins either aggregate to the point of becoming hydrophobic and are therefore unable to be degraded by the lysosome or that there are no sufficient acidified lysosomes that are readily available for their degradation (Lee J. H. et al., 2022; Lee S. E. et al., 2022). Once expelled from the neuron, Aβ42 aggregates and clumps together, resulting in the insoluble senile plaques that characterize the disease (Mucke and Selkoe, 2012). The extracellular presence of these plaques triggers an immune response from microglia and astrocytes, both of which begin producing ROS and reactive nitrogen species (RNS) against the unfamiliar, proteinaceous material (Heales et al., 2004; Kitazawa et al., 2004; Hickman et al., 2008). This RO/NS production appears to bring about several consequences. Indeed, studies have revealed that this increase produces a pro-inflammatory state within the extracellular region which contributes toward cytotoxicity, and anti-amyloid therapies may therefore be a poor choice in combating AD symptoms (Patel et al., 2005; García-Bueno et al., 2008; Jin et al., 2008; Huang et al., 2020). Additionally, according the amyloid cascade hypothesis, the manifestation of this pro-inflammatory state contributes toward the hyperphosphorylation of the microtubule-associated protein, tau, thereby leading to the appearance of NFTs (Hardy and Selkoe, 2002). However, other studies have indicated that it may be plausible that tauopathies arise prior to the formation of senile plaques (Braak et al., 2011; Theofilas et al., 2018). Indeed, tauopathies may also arise due to mutations in the gene that encodes for tau; *MAPT* and tauopathies that arise due to the initial appearance of Aβ42 aggregation are known as “secondary tauopathies” (Iovino et al., 2015; Leveille et al., 2021). Regardless of the order of molecular events unfolding, the appearance of both senile plaques and NFTs are commonplace in brains of AD patients (Näslund et al., 2000; Ando et al., 2014) and therefore deserve equal levels of attention. Since tau is responsible for microtubule assembly and stabilization (Binder et al., 1985; Page et al., 2010), its hyperphosphorylation will result in its dissociation from microtubules; causing the formation of oligomeric tau, the loss of microtubule stability and therefore a loss in microtubule-dependent transport (Iqbal et al., 2009) thereby impacting axonal transport (Page et al., 2010). Additionally, increased levels of oligomeric tau have been shown to disrupt the ETC by co-localizing with complex V, leading to a diminishment of ATP production (David et al., 2005). The overall loss of microtubule-dependent transport coupled with mitochondrial energy disruption has been shown to reduce synaptic activity as well as organelle transport throughout the neuron (Lasagna-Reeves et al., 2011). As a result, neurons undergo cell death and form NFTs, a key hallmark of tauopathies, a group of neurodegenerative disease that is often seen in AD patients (Braak and Braak, 1991; Jack et al., 2010), but has also been reported to develop alongside Parkinson’s disease or even independently (Dickson, 2010; Wills et al., 2010).

Evidence suggests that the presence of senile plaques and NFTs are in fact the end result of disrupted proteostasis. Senile plaques in particular have been suggested to represent an attempt made by the cell to expel harmful proteinaceous components to prevent their interactions with mitochondria and thereby decreasing further ROS generation within the cell (Nixon et al., 2005; Abramov et al., 2009; Serrano-Pozo et al., 2011) whereas NFTs represent a ‘left behind’ remnant after the oligomerization of hyperphosphorylated tau has induced neuronal cell death (Braak and Braak, 1991; Min et al., 2010; Page et al., 2010). Indeed, the soluble, intracellular Aβ42 and hyperphosphorylated tau oligomers, and not their aggregated, insoluble counterparts, appear to cause the decrease in synaptic transmission and the eventual death of neurons observed in AD, as these effects appear to occur prior to the formation of senile plaques and NFTs (Mucke et al., 2000; Manczak et al., 2006; Cirrito et al., 2008; Lasagna-Reeves et al., 2011; Polito et al., 2014).

### 7.2. Parkinson’s disease: ROS, α-synuclein and mitochondrial clearance failure

Parkinson’s disease (PD) is histologically characterized by the presence of intracellular Lewy bodies; α-synuclein-containing protein inclusions that are particularly located within the cell bodies of dopaminergic neurons of the substantia nigra (Spillantini et al., 1998; Kouli et al., 2018; Kavuri et al., 2020). Motor symptoms associated with the disease, such as severe tremors, rigidity and slowed movement, are due to the dopamine toxicity (Sveinbjornsdottir, 2016) or failure of mitochondrial clearance through mitophagy (Chinta et al., 2010; Moskal et al., 2020). Additionally, Braak et al. (2004) have demonstrated the pattern of neuronal degeneration in other regions of the brain as PD progresses, such as the brain stem and hippocampus, thereby demonstrating additional characteristic symptoms of the disease, some of which overlap with AD symptoms such as memory loss and mood changes (Braak et al., 2004).

Similar to AD, PD manifests in either a genetic or sporadic manner, with genetic manifestations being due to either autosomal dominant or recessive mutations (Reviewed in Breydo et al., 2012), which distinguish themselves from one another at the molecular level despite leading to similar symptoms. The autosomal dominant form of PD is due to several possible mutations in the *SNCA* gene. As a result, α-synuclein; the protein expressed by *SNCA* transcription, is expressed with a misfolded conformation (Polymeropoulos et al., 1997; Le and Appel, 2004). Natively expressed α-synuclein plays an important role in synaptic vesicle transmission and recycling (Cheng et al., 2011). Additionally, α-synuclein regulates the synthesis of dopamine by interacting with and inhibiting tyrosine hydroxylase; a rate-limiting enzyme for the synthesis of dopamine (Perez et al., 2002; Di Maio et al., 2016). Since dopamine is highly reactive and causes ROS production levels must be properly maintained, especially in dopaminergic neurons (Kao et al., 2002). For this reason, dopamine is sequestered into vesicles by vesicle monoamine transporter 2 (VMAT2) such that its reactive potential is removed from the cytoplasmic environment (Kao et al., 2002; Lotharius and Brundin, 2002; Ulusoy et al., 2012).

However, misfolded, oligomeric α-synuclein, causes dysfunctional dopamine storage. Misfolded α-synuclein expression has been demonstrated to be accompanied by reduced levels of VMAT2 and increased cytoplasmic dopamine levels (Lotharius et al., 2002; Guo et al., 2008; Zhou et al., 2011). As a result, dopamine remains in the cytoplasm and reacts with oxygen to produce ROS. This ROS generation causes subsequent mitochondrial stress, thereby increasing ROS generation further and finally leading to the death of the dopaminergic cell (Whitworth et al., 2005; Ulusoy et al., 2012). Additionally, it has been shown that misfolded α-synuclein co-localizes with complex I of the mitochondrial ETC and thereby interferes with normal ATP generation, leading to the increased production of ROS as well as increasing the likelihood of inducing apoptosis (Chinta et al., 2010; Di Maio et al., 2016; Ding et al., 2018). Other factors contributing toward the genetic expression of PD have been reviewed and are discussed by Nagatsu et al. (2019). It is therefore clear that the role of α-synuclein in regulating dopamine production, especially within the dopaminergic neurons, is key in controlling and dampening its damaging potential toward the cell (Chu and Kordower, 2007). The degradation of wild-type α-synuclein appears to occur primarily through chaperone-mediated autophagy (CMA) which links cargo with LAMP2a for degradation by the lysosome (Mak et al., 2010). However, it has been reported that CMA as well as the UPS are both blocked in the brains of PD patients (Mcnaught et al., 2002; Massey et al., 2006; Ebrahimi-Fakhari et al., 2011). Indeed, Martinez-Vicente et al. (2008) have shown that the post-translational modification of wild-type α-synuclein by dopamine leads to a decreased clearance via lysosomes (Martinez-Vicente et al., 2008; Tang et al., 2015) thereby contributing toward their accumulation and the formation of Lewy bodies. Additionally, a study conducted by Ejlerskov et al. (2015) involved the deletion of an important component of the interferon. The result was the appearance of PD-associated symptoms, such as cognitive impairments and motor deficiencies in mice, as well as the appearance of Lewy bodies, all of which appeared to be accompanied with changes in autophagy as well as receptors p62 and NBR1, confirming the involvement of macroautophagy in PD pathology (Ejlerskov et al., 2015). Taken together, it becomes clear that oligomeric α-synuclein causes a dysfunction in autophagy flux as well as decreased ATP generation by mitochondria, thereby contributing toward cell death onset.

The misfolded structure of mutated α-synuclein makes it highly aggregate-prone and the risk of forming insoluble intracellular Lewy bodies is increased as the degradation capacity decreases (Spillantini et al., 1998; Zhou et al., 2011; Ejlerskov et al., 2015). Lewy bodies are composed primarily of α-synuclein but have also been shown to consist of other materials such as PINK1 and Parkin, as well as iron and many other constituents (Baba et al., 1998; Wakabayashi et al., 2013). The formation of Lewy bodies has been shown to lead to a decrease in tyrosine hydroxylase, therefore leading to a decrease in dopamine-dependent neurotoxicity in the substantia nigra (Mori et al., 2006) as well as minimizing the reactive activities of various components and therefore offers neuroprotective benefits to the neuron (Wakabayashi et al., 2006). However, Lewy bodies may act as neuronal obstructions that hinder axonal transport (Katsuse et al., 2003), thereby leading to decreased synapse firing and contributing toward the cognitive and motor impairment associated with the disease (Volpicelli-Daley et al., 2011; Games et al., 2013; Hassink et al., 2018). The autosomal recessive form of PD is a result of mutations in *PARK2* and *PARK6* genes (Kitada et al., 1998). PINK1, translated from *PARK6* (Valente et al., 2004) and Parkin, translated from *PARK2* work together to maintain the pool of healthy mitochondria within the neuron through mitophagy. Indeed, PINK1 has been shown to bear a mitochondrial targeting sequence and is thereby able to be recruited to mitochondria at basal levels. However due to the manner in which PINK1 is cleaved, it is later released into the cytosol where it is cleared by the UPS (Jin et al., 2010; Yamano and Youle, 2013). Should the mitochondria be depolarized or exposed to cellular stress, PINK1 will remain on the mitochondrial membrane in its uncleaved form where it acts as a receptor for Parkin; an E3 ligase, which aids in the degradation of depolarized mitochondria by acting as a receptor for autophagosomes (Narendra et al., 2008; Lazarou et al., 2012; Okatsu et al., 2013) (see section 6 and Figure 2). As such, the combined function of both PINK1 and Parkin are shown to enable successful mitophagy, thereby contributing toward the elimination of depolarized mitochondria and the ROS that would have been produced.

The genetic failure of the PINK1/Parkin relationship results in mitophagy dysfunction and the accumulation of depolarized mitochondria within neuronal cytoplasm. Patients with these recessive mutations have been shown to display symptoms that are clinically indistinguishable from sporadic PD aside from having an earlier onset (Yang and Tiffany-Castiglioni, 2005; Houlden et al., 2012; Chai and Lim, 2013). The exact reason why mitophagy dysfunction causes PD symptoms remains unclear, however due to the high mitochondrial actions required in the substantia nigra for the purpose of dopamine release, it stands to reason that although this dysfunction manifests across the entire brain, the substantia nigra is the first region to suffer from degeneration (Reviewed in Surmeier et al., 2017). In addition to AD, PD has been described primarily as a sporadic disorder (Gasser, 2009; Houlden et al., 2012). Sporadic PD has been suggested to be contributed toward and manifests as a result of exposure to environmental toxins, an increased aggregation of misfolded proteins or increased systemic oxidative stress resulting in the increased production of ROS (Schapira et al., 1990; Yang and Tiffany-Castiglioni, 2005; Parihar et al., 2008; Okatsu et al., 2013; Di Maio et al., 2016). Additionally, Studies using MPTP (1-methyl-4-phenyl-1,2,3,6-tetrahydropyridine), the metabolites of which are known to inhibit mitochondrial complex I, have demonstrated the effects felt by dopaminergic neurons given increased oxidative damage. This appears to be similar to the pathological effects of misfolded α-synuclein, however the use of MPTP does not seem to induce the formation of Lewy bodies (Kowall et al., 2000; Shimoji et al., 2005). Indeed, Cleeter et al. (1992) have used MPTP in animal models and found that the resulting effects on dopaminergic neurons of the substantia nigra were similar to those observed in autosomal dominant PD. It has been shown that the induction of autophagy by rapamycin protected mice against MPTP induced dopaminergic neuron loss (Zhang et al., 2017; Pupyshev et al., 2019). Additionally, studies using MPTP have also shown that there is an increase in *PARK1* activity. This increased *PARK1* activity leads to increased expression of mutant α-synuclein and therefore an increase in cytoplasmic dopamine as described in the case of autosomal dominant PD (Vila et al., 2000) and provides crucial insight into the study of PD.

### 7.3. Huntington’s disease: soluble and aggregated cargo causing distinct dysfunction

Huntington’s disease (HD) is an autosomal dominant neurodegenerative disorder caused by mutations in the huntingtin (*HTT*) gene and characterized by the initial loss of neurons along the striatum that slowly progresses into other brain regions including the cerebral cortex and hypothalamus (Vonsattel et al., 1985; DiFiglia et al., 1997; Tebbenkamp et al., 2011). As a result, the clinical symptoms of HD include dysfunctional motor capabilities (Dragatsis et al., 2000; Guo et al., 2012) followed by cognitive impairment (Bäckman et al., 1997; Montoya et al., 2006; Vinther-Jensen et al., 2014). Brains of affected patients have been shown to contain intranuclear inclusion bodies and dystrophic neurites (Davies et al., 1997; Iwata et al., 2009). These inclusions contain mutant huntingtin (mHtt) proteins with polyglutamine (polyQ) repeats at the NH2-terminal, demonstrating the detrimental role of this protein in the pathogenesis of the disease. In healthy cells, wild-type Htt appears to be localized in the cytoplasm and associates with organelles such as mitochondria, synaptic vesicles and cytoskeletal components (Li et al., 2001; Orr et al., 2008; Morfini et al., 2009; Shirendeb et al., 2011). As such, Htt has been suggested to play important roles in anti-apoptotic signaling, post-synaptic transmission and protein trafficking (Reviewed in Schulte and Littleton, 2011), however, the exact role remains mysterious.

Molecularly, HD has been shown to be as a result of a mutation that causes the expansion of polyQ repeats at the NH2-terminal of the Htt protein (MacDonald et al., 1993). The number of polyQ repeats vary according to the individual and appear to correlate with the rate at which the disease progresses (Arrasate et al., 2004). Studies have demonstrated that the NH2-terminal expansions of mHtt bear sites that are selectively cleaved by proteases such as caspases and calpains (Kim et al., 2001; Wellington et al., 2002; Tebbenkamp et al., 2011, 2012). The result is the production of mHtt fragments that have been shown to be aggregate-prone and therefore toxic to the cellular system. Indeed, it appears that it is these soluble, toxic proteins that are responsible for many of the cellular disruptions that arise in the case of HD such as dysfunctional autophagy cargo recognition, diminished microtubule-dependent transport of vesicles and inappropriate mitochondrial fission and therefore dysfunctional mitophagy (Martinez-Vicente et al., 2010; Shirendeb et al., 2011, 2012). The Increased production of mHtt suggests that there would be a downregulated production of natively-folded wild-type Htt and therefore a deficit in the regular necessary activities of Htt. The aggregate-prone nature of mHtt causes a disruption to the proteostatic balance of the cell unless levels are reduced. Iwata et al. (2005) have demonstrated that there is no clear autophagy activity visible within the nucleus. However, the nucleus has been found to be make use of the UPS to facilitate degradation, thereby implicating the nucleus as being wholly dependent upon the UPS for protein degradation (Iwata et al., 2005; Chow et al., 2012; Juenemann et al., 2013; Bhat et al., 2014). Consequently, highly aggregated mHtt has been found to inhibit the UPS. Despite its final nuclear destination, the fact that oligomeric mHtt is known to co-localize with key cellular structures, makes it clear that it does exist within the cytoplasm for some time. It stands to reason therefore that it is vital for autophagy and the UPS operating together to minimize oligomeric mHtt prior to its aggregation and co-localization with the nucleus. As is the case in AD and PD, mHtt monomers aggregate to the point where they are no longer able to be degraded by the proteasome and must therefore be degraded through autophagy. Indeed, the use of rapamycin and rilmenidine, respectively have been found to induce autophagy and lead to enhanced clearance of mHtt (Berger et al., 2006; Rose et al., 2010). However, it has been proposed that mHtt associates with p62 in such a way that there is improper cargo recognition by the autophagosome, causing a slower turnover of mHtt through autophagy (Stolz et al., 2014).

Furthermore, mHtt has been found to associate with the outer mitochondrial membrane, translocating to the inner membrane where it interferes with the ETC, thereby disrupting ATP generation and increasing ROS generation (Orr et al., 2008; Shirendeb et al., 2011). Additionally, aggregated mHtt has been shown to localize along the axon, leading to an inhibition of axonal transport (Li et al., 2001; Morfini et al., 2009). This leads to a decrease in mitochondrial trafficking and thus reduces the number of mitochondria present at pre-synaptic terminals. As a result, mitochondria are no longer able to reach ATP-demanding regions -such as pre-synaptic terminals- resulting in a reduction in neurotransmitter release (Orr et al., 2008; Shirendeb et al., 2012). Additionally, HD has been shown to exhibit physiological changes in glutamatergic signaling (Faideau et al., 2010; Miller et al., 2012) as well as NMDAR signaling (Beal et al., 1991; Lee et al., 2006; Dau et al., 2014), both of which contribute toward excitotoxicity expected in the brain.

Taken together, oligomeric mHtt causes disruptions in the proteostatic balance, leading to an accumulation of dysfunctional mitochondria, decreased autophagy and excitotoxicity. Aggregated mHtt appears to disrupt axonal transport systems, thereby inhibiting synaptic transmission. The relationship between various rates of autophagy and subsequent clearance of diffuse or possibly aggregated mHtt is, however, largely unknown, and deserves further study.

## 8. The autophagy triad

**Figure 6:**
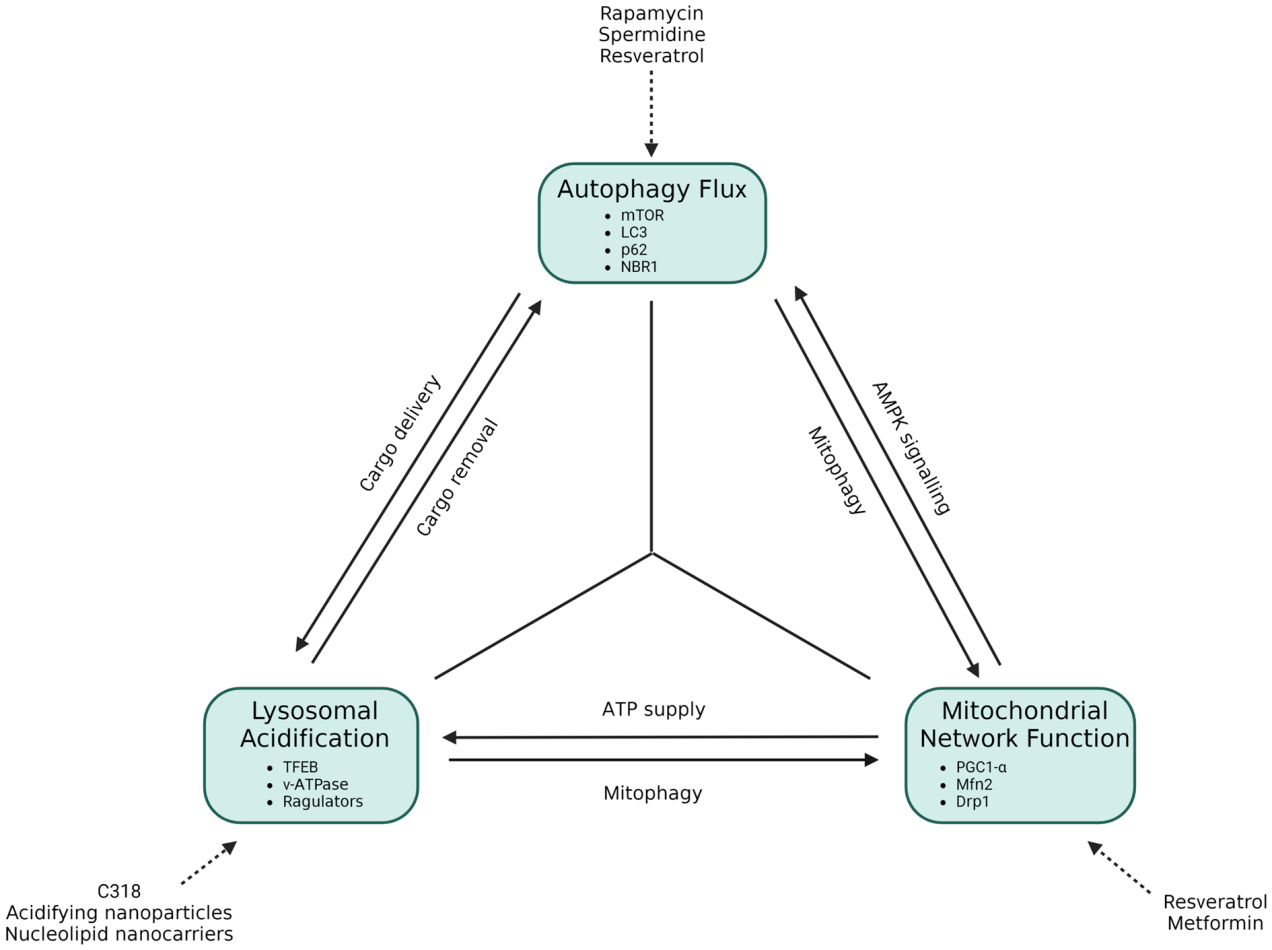
The functional autophagy triad. Autophagy flux requires the availability of acidified lysosomes, which are dependent on ATP output through oxidative phosphorylation. Mitochondria, in turn, are dependent on the amino acid by-products brought about from autophagy, but also require the autophagy process to eliminate dysfunctional mitochondria through mitophagy. Autophagy initiation is also dependent upon the health of mitochondria, as this determines autophagosome initiation activity through AMPK signaling. In this way an interdependent, triangular relationship between these components becomes apparent, which aids in the cellular stress response and cell survival. If one of these components is disrupted or dysfunctional, a direct or indirect knock-on effect on the next component emerges, and in so doing causes cellular stress that may contribute to neurodegenerative disease.

It becomes clear that the soluble oligomers are problematic and reducing their expression at a cellular level is key to restoring cellular homeostasis. The autophagy pathway relies heavily upon the availability of acidified lysosomes which in turn only achieve a sufficient acidification status through the ATP produced through oxidative phosphorylation. Mitochondria in turn depend on the substrates released from the autophagy pathway, i.e., glucogenic and ketogenic amino acids, following cargo degradation. Here we will collectively term this cross talk the “autophagy triad” (Figure 6).

Autophagy activity is crucial in providing substrates for oxidative phosphorylation, and mitophagy is important for the maintenance of a healthy mitochondrial network by eliminating depolarized mitochondria (Burman et al., 2017; Rana et al., 2017). Furthermore, loss of autophagy cargo recognition has been shown to result in an increase in aggregate formation (Bhattacharjee et al., 2022) and contributes toward neurodegenerative disease manifestation. Indeed, a study investigating a p62 mutant in amyotrophic lateral sclerosis (ALS) showed that p62 behavior was changed in such a way that autophagy activity and mitochondrial respiration were both significantly decreased (Bartolome et al., 2017). Additionally, studies in which PINK1 and Parkin have been knocked out, respectively have demonstrated a decrease in mitochondrial activity, highlighting the key role of mitophagy in maintaining mitochondrial health (Song et al., 2017; Zhi et al., 2019).

Highlighted in section 6, mitochondria are dynamic organelles, undergoing constant fission and fusion events in response to the metabolic requirements of the cell. Fusion events occur through mitofusin-2 (Mfn2) and these events are shown to result in the increase of ATP, with cases of hyperfusion occurring due to a loss in ETC complex IV activity (Rolland et al., 2013). Fission events on the other hand, occur primarily through the dynamin-1-like protein (Drp1), which is responsible for separating the mitochondria in response to metabolic stress for the purpose of eliminating harmful, ROS producing mitochondria from the system (Rana et al., 2017; Fonseca et al., 2019). Indeed, Fang et al. (2019) have shown an accumulation of dysfunctional mitochondria in the neurons of AD patients, demonstrating mitophagy dysfunction early in disease progression (Fang et al., 2019). It becomes clear therefore that mitochondrial network health is vital for the maintenance of mitochondrial quality and for enabling acidification of lysosomes (Yagi et al., 2021; Huang et al., 2022).

Studies in which ATP6AP2, a key component of the lysosomal acidification machinery, had been knocked down in *Drosophila* and mouse models demonstrated an increase in autophagosomes along with diminishments in cognitive performance (Dubos et al., 2015; Hirose et al., 2019). Likewise, the loss of lysosomal acidity has been shown to be accompanied by an accumulation of autophagosomes as a result of a decline in autophagy flux (see section 3). The restoration of lysosomal acidification through novel nanoparticles and small molecules has been shown to decrease the numbers of autophagosomes present in the cell, demonstrating an increase in autophagy flux while also enhancing the presence of healthy mitochondria, likely through the induction of mitophagy (Vest et al., 2022; Brouillard et al., 2023; Zeng et al., 2023).

Dysfunction within any of these three entities may hence contribute toward the onset of neurodegenerative disease (Figure 6). Likewise, it is clear that therapeutic interventions that lead to the increased activity of one entity (as reviewed by Lumkwana et al., 2017; Lo and Zeng, 2023) will have subsequent knock-on effects that will enhance the functioning of the other.

## 9. Concluding remarks

AD, PD and HD constitute the most prevalent neurodegenerative diseases. Whilst all three have been shown to exhibit genetic inheritance, it is concerning to note that sporadic AD and PD are commonplace in individuals within aging populations and present without genetic factors (Greenamyre and Hastings, 2004; Alzheimer’s Association, 2022). As such, the incidence of AD and PD is of increasing concern as it reflects lifestyle as well as the environmental factors as contributing causes for neurodegenerative disorders (Winklhofer and Haass, 2010; Genuis and Kelln, 2015; Kell and Pretorius, 2015; Xu et al., 2016; Colacurcio and Nixon, 2017; An et al., 2018).

Arrasate et al. (2004) have demonstrated that a greater level of mHtt inclusion bodies is positively correlated with greater cell survival. In PD, Lewy bodies are shown to consist of iron as well as misfolded α-synuclein. By removing misfolded α-synuclein from the cytoplasm, it may be that there is less dopamine dysregulation, whereas removing iron prevents a reaction with dopamine that would result in the production of ROS (Castellani et al., 2000; Lotharius et al., 2002; Ulusoy et al., 2012). Aβ oligomers are released to the extracellular microenvironment, by a mechanism which is poorly described, but is likely to involve mitochondria-associated ER membrane, where they aggregate into senile plaques, thereby potentially eliminating the harmful oligomers from the cytoplasm (Ray et al., 2010; Nilsson et al., 2013; Bhattacharyya et al., 2021). However, prior to the formation of senile plaques, these proteins have been shown to trigger an immune response which leads to neuroinflammation in the extracellular milieu. Additionally, their presence in the extracellular millieu enables them to interact with NMDA receptors in such a way that induces excitotoxicity through excessive Ca^2+^ entry into the neuron (Kitazawa et al., 2004; Shankar et al., 2007; Goussakov et al., 2010; Ma et al., 2014). The immune response in particular may be exacerbated by increased hydrophobicity leading to the formation of senile plaques, thereby further increasing the neurotoxicity produced. Inclusion bodies are the hallmark characteristic used to histologically identify and distinguish between neurodegenerative diseases. They are composed of oligomeric forms of misfolded proteins which increase in hydrophobicity as they aggregate over time, forming the insoluble inclusion bodies that are deposited in regions of the brain that correspond with the symptoms of the disease at later stages of the disease. In addition to the characteristic oligomers, inclusion bodies have also been shown to consist of p62 and ubiquitin, demonstrating that autophagy plays a critical role in the formation of insoluble aggregations (Hara et al., 2006; Komatsu et al., 2007; Jackson et al., 2017). It is therefore plausible that inclusion bodies are not the root cause of neurotoxicity *per se*, but rather that they arise due to autophagy dysfunction brought about by the effects of the oligomeric constituents of the inclusion bodies that cause damage to intracellular organelles (Figure 6).

It is however of vital importance that their production be minimized in a timely manner so as to avoid disruptions of the mitochondrial ETC (Hansson Petersen et al., 2008; Orr et al., 2008; Parihar et al., 2008; Rao et al., 2014; Di Maio et al., 2016) or ER stress (Timmins et al., 2009; Gupta et al., 2010), that can lead to increased intracellular Ca^2+^ levels (Yu et al., 2009; Affaticati et al., 2011; Zündorf and Reiser, 2011; McBrayer and Nixon, 2013), both of which contribute toward a cytotoxic environment; therefore driving the likelihood of cell death onset. Additionally, inducing autophagy with mTOR inhibitors such as rapamycin has been shown to decrease the availability of these proteins (Caccamo et al., 2010; Malagelada et al., 2010; Spilman et al., 2010) and other inducers of autophagy such as spermidine, resveratrol and metformin, to name a few, have also been shown to result in reduced levels of oligomers and thus contributing toward the survival of the cell (Eisenberg et al., 2009; Shi et al., 2012; Ai et al., 2015; Pietrocola et al., 2015; Wang et al., 2018) although this should be performed with caution so as to avoid – albeit unlikely – autosis onset. It becomes clear therefore that the misfolded conformations of these key proteins are what drives cytotoxicity as their effects occur prior to the appearance of inclusion bodies, an important consideration when developing effective therapies against neurodegenerative disease in order to avoid consequential neuroinflammation.

The mild hydrophobicity of soluble oligomers creates a challenge to the autophagy system which increases autophagosome production as a means of increasing flux as a stress response to decrease the protein load (Nixon et al., 2005; Fujita et al., 2008a; Menzies et al., 2015; Bordi et al., 2016). Although basal autophagosome counts in neurons are generally low, treatment with lysosomal inhibitors such as bafilomycin A1 or chloroquine cause an accumulation of autophagosomes, lysosomes and autolysosomes demonstrating that neuronal autophagy flux is in fact very high (Boland et al., 2008; du Toit et al., 2018a). Regardless, patients still suffer from symptoms of neurodegeneration. This is likely because there is an overproduction and oligomerization of misfolded proteins, generated at a greater rate than the rate of their degradation through autophagy (Martinez-Vicente et al., 2010; Wu et al., 2015; Bordi et al., 2016). A possible reason for this may be due to the interaction between oligomers and mitochondria, resulting in decreased ATP output (Kim et al., 2001; Manczak et al., 2006; Hansson Petersen et al., 2008; Chinta et al., 2010; Di Maio et al., 2016). Since lysosomes require ATP to be reacidified after the degradation of cargo (Forgac, 2007; Zoncu et al., 2011), the decreased availability of ATP will lead to a decrease in protein degradation in spite of cargo sequestration by autophagosomes (Figure 6). Indeed, Lee J. H. et al. (2022) and Lee S. E. et al. (2022) have recently demonstrated the importance of acidified lysosome pool size in the context of AD and neuronal health (Colacurcio and Nixon, 2017; Lee J. H. et al., 2022). Additionally, this will result in dysfunctional mitophagy, leading to the accumulation of dysfunctional mitochondria and their continued production of ROS and pro-apoptotic signaling molecules (Narendra et al., 2008; Orr et al., 2008; Zhang and Ney, 2009). Taken together, it is plausible that the stress produced by mitochondria and the subsequent loss of lysosomal acidification lies at the heart of neurodegeneration as autophagy flux is dependent upon adequate mitochondrial output (Figure 6). It is important to note that these interactions occur intracellularly but create extracellular stressors that lead to knock-on effects to surrounding cells. Increasing the autophagy flux seems to be the gold standard to decreasing ND toxicity, however, as it is known that dysfunctional mitochondria do continue to accumulate during the pathogenesis of the disease, it is conceivable that increasing autophagy flux will work to a degree but reach a point where it is no longer effective as the lysosomal pool becomes increasingly deacidified. If our suggested “autophagy triad” is relevant (Figure 6), then it may be the case that inducing mitochondrial biogenesis or mitochondrial fusion, through treatments such as resveratrol or metformin, might be more beneficial than previously anticipated, as these will contribute toward lysosomal acidification and subsequently autophagy flux that effectively aids in the clearance of depolarized mitochondria as well as NDD proteins. This notion would strongly support a combined therapy that makes use of upregulated mitochondrial biogenesis paired with autophagy flux induction, therefore impacting lysosome function and subsequent mitophagy and proteinaceous cargo capacity. This deserves further study.

## Author contributions

SdW conceptualized, wrote the manuscript, and developed the figures. BL conceptualized. BL and RT edited the manuscript. All authors contributed to the article and approved the submitted version.

## Conflict of interest

The authors declare that the research was conducted in the absence of any commercial or financial relationships that could be construed as a potential conflict of interest.

## Publisher’s note

All claims expressed in this article are solely those of the authors and do not necessarily represent those of their affiliated organizations, or those of the publisher, the editors and the reviewers. Any product that may be evaluated in this article, or claim that may be made by its manufacturer, is not guaranteed or endorsed by the publisher.
